# Mechanisms and clinical application of photobiomodulation in pain therapy: a mini review

**DOI:** 10.3389/fpain.2026.1920821

**Published:** 2026-08-18

**Authors:** Yuxuan Ren, Weiwei Li, He Miao, Yushan Chen, Xiaowei Chen

**Affiliations:** Health Science Center, Ningbo University, Ningbo, Zhejiang, China

**Keywords:** analgesia, green light, near-infrared, pain, photobiomodulation, red light, visual-light exposure

## Abstract

Chronic pain remains a major clinical challenge, creating a need for safe, non-pharmacological interventions. This narrative mini-review examines two light-based approaches that differ in their sites of action and treatment parameters: visual-light exposure and local red or near-infrared light-emitting diode photobiomodulation. Visual-light exposure acts through retinal and central neural circuits, including pathways involving the ventrolateral geniculate nucleus, dorsal raphe nucleus, periaqueductal gray, medial secondary visual cortex, and anterior cingulate cortex. Its effects are wavelength- and context-dependent. Green light has generally produced antinociceptive effects in preclinical studies and preliminary clinical benefits in migraine, whereas visual exposure to red light has predominantly been associated with increased pain. White light can either facilitate or suppress pain depending on irradiance, exposure duration, circadian timing, and clinical context. By contrast, locally applied red or near-infrared LEDs have reduced pain in several preclinical models and limited clinical studies through peripheral opioid signaling, modulation of nitric oxide and nociceptor sensitization, and anti-inflammatory and antioxidant effects. The opposing effects of visual and local red light indicate that wavelength alone does not determine the pain response; exposure route, target tissue, dose, timing, and baseline pain state are also critical. Although visual-light interventions and local LED PBM appear promising as adjuncts to conventional treatment, the evidence remains limited by heterogeneous protocols, small clinical samples, and incomplete dosimetric reporting. Future studies should use standardized parameters, rigorous controls, and condition-specific protocols to establish efficacy and identify patients most likely to benefit.

## 1 Introduction

In 2020, the International Association for the Study of Pain redefined pain as “an unpleasant sensory and emotional experience associated with, or resembling that associated with, actual or potential tissue damage” (1). Pharmacological options include acetaminophen, non-steroidal anti-inflammatory drugs, adjuvant analgesics, and opioids, depending on the pain condition and severity. However, long-term use may be limited by inadequate efficacy and adverse effects. Opioids carry risks of tolerance, dependence, constipation, and respiratory depression, while non-steroidal anti-inflammatory drugs may cause gastrointestinal, renal, and cardiovascular complications (2, 3). Safe and effective non-pharmacological alternatives are therefore needed, especially for chronic pain.

Low-level laser therapy (LLLT) was first investigated by Endre Mester in the 1960s (4). As non-laser light sources, including light-emitting diodes (LEDs), became more widely used, the broader term photobiomodulation (PBM) was adopted (5, 6). Following the nomenclature proposed by Anders, Lanzafame, and Arany, PBM refers to the therapeutic application of non-ionizing visible or near-infrared light-generally at low output powers, commonly described as up to 500 mW-to induce nonthermal photophysical and photochemical responses through interactions with endogenous chromophores (6).

The scope of PBM remains a matter of terminology. Conventional PBM generally refers to local irradiation intended to act directly on tissue chromophores. However, some reviews have also included visual-light exposure, which primarily acts through retina photoreception and downstream neural or circadian pathways (7). These modalities are not assumed to share the same mechanisms although both use visible light. Accordingly, this review treats visual-light exposure and local LED-based PBM as related but mechanistically distinct interventions.

The importance of this distinction is illustrated by the effects of red light. Visual exposure to red light has generally increased pain-related responses in experimental studies (8–11), whereas local irradiation with red or near-infrared LEDs has reduced pain and inflammation in several preclinical and clinical settings (12–18). White light also has context-dependent effects, facilitating nociception after brief bright-light exposure or circadian disruption but producing analgesia under some prolonged morning-treatment protocols (19, 20). Green visual light has shown predominantly antinociceptive effects, although the clinical evidence remains preliminary (21–27). Thus, wavelength alone is insufficient to predict the direction of the pain response. Exposure route, irradiance or illuminance, duration, circadian timing, target tissue, and baseline pain state must also be considered.

This narrative mini-review compares the effects and mechanisms of retina-mediated visual-light exposure with those of locally applied red and near-infrared LEDs. It also evaluates the limitations of the current evidence and discusses the potential role of these interventions as adjuncts in pain management.

## 2 Literature search and review scope

This narrative mini-review was informed by searches of PubMed for English-language publications, primarily from 1992 through 2026. Search terms included combinations of “photobiomodulation,” “light-emitting diode,” “white light,” “bright light,” “green light,” “red light,” “near-infrared light,” “pain,” “nociception,” “antinociception,” and “analgesia.” Original preclinical and clinical studies were considered if they evaluated pain-related outcomes after retina-mediated visual-light exposure or local red/near-infrared LED treatment. Foundational older publications and mechanistically relevant studies identified from reference lists were also considered. Studies focused primarily on ultraviolet treatment, ionizing radiation, or high-intensity laser therapy were excluded. Because this was a narrative rather than systematic review, study selection was not conducted according to PRISMA, and no formal meta-analysis or risk-of-bias assessment was performed.

## 3 Retina-mediated effects of visual-light exposure

**Figure 1:**
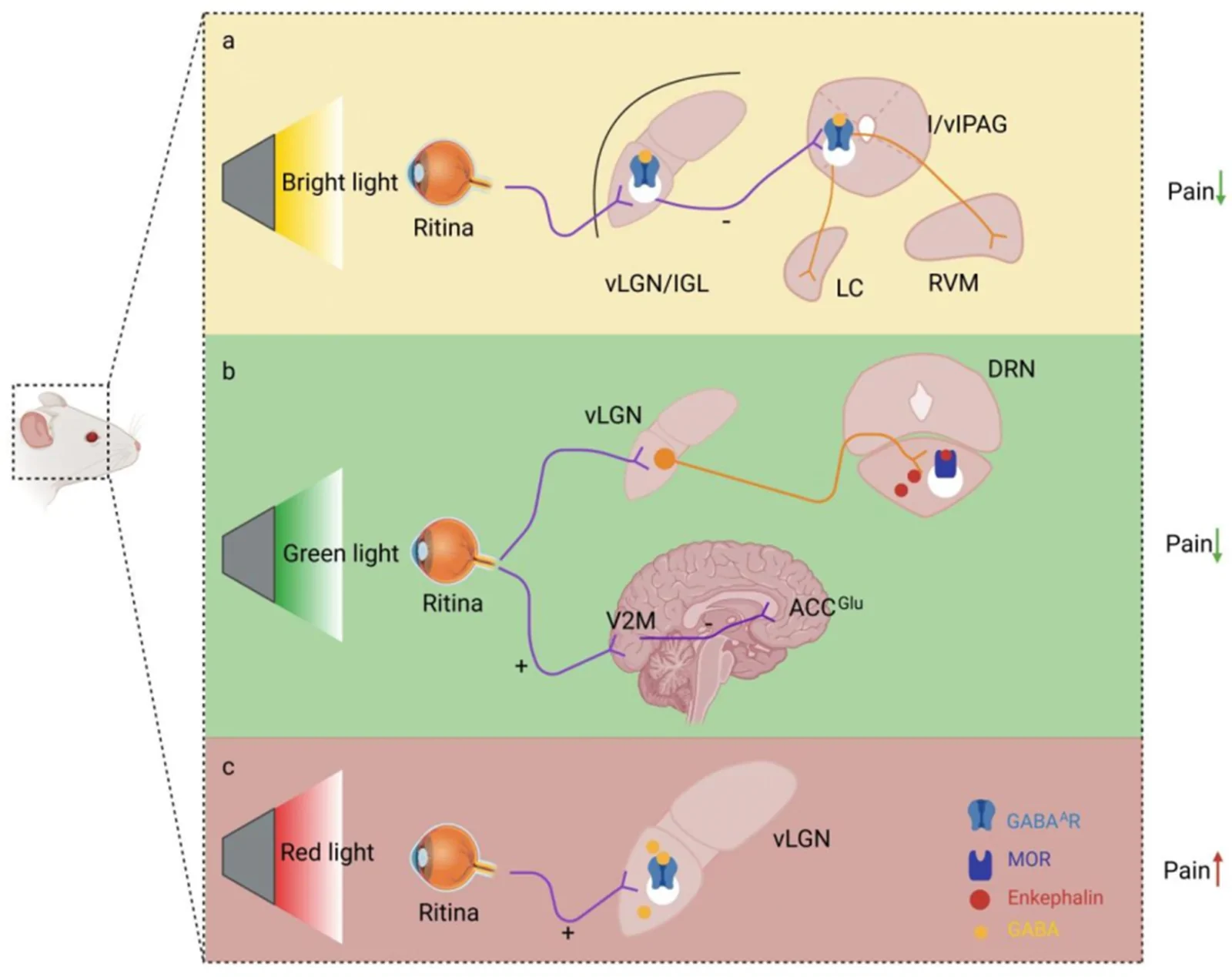
Visual circuits mediating wavelength- and context-dependent modulation of pain. **(a)** Repeated bright-light exposure activates retinal ganglion cell projections to the ventrolateral geniculate nucleus/intergeniculate leaflet (vLGN/IGL). Gamma-aminobutyric acid (GABA)-ergic vLGN/IGL projections inhibit GABAergic neurons in the lateral/ventrolateral periaqueductal gray (l/vlPAG), thereby influencing downstream locus coeruleus (LC) and rostral ventromedial medulla (RVM) circuits and producing antinociception. **(b)** Green light activates retinal inputs to enkephalinergic vLGN neurons projecting to the dorsal raphe nucleus (DRN) and recruits a medial secondary visual cortex-anterior cingulate cortex (V2M-ACC) pathway that suppresses ACC excitatory activity. **(c)** Red light may facilitate pain via vLGN GABAergic neuron activation.

Visual-light exposure differs from local tissue PBM because its pain-modulatory effects are initiated predominantly by retinal photoreception and transmitted through central neural and circadian pathways. Preclinical studies have implicated circuits involving the ventrolateral geniculate nucleus (vLGN), intergeniculate leaflet (IGL), periaqueductal gray (PAG), locus coeruleus (LC), rostral ventromedial medulla (RVM), olivary pretectal nucleus (OPt), dorsal raphe nucleus (DRN), medial secondary visual cortex (V2M), and anterior cingulate cortex (ACC). Different wavelengths, exposure schedules, and pain states may recruit distinct or even opposing circuits, as summarized in Figure 1.

### 3.1 White light

Okamoto et al. found that bright light activates trigeminal nociceptive pathways via an intraocular route through the olivary pretectal nucleus, superior salivatory nucleus, and parasympathetic signaling (28, 29). Martenson et al. showed that bright light (18,000 lux, 30 s) acts through the olivary pretectal nucleus (not thalamus or trigeminal ganglion) to activate RVM “ON-cells” and suppress “OFF-cells”, biasing pain modulation toward pronociception (30). Nocturnal light disrupts circadian rhythms and melatonin, enhancing pain; dim night light in animals induced cold hyperalgesia, mechanical allodynia, PAG *μ*-opioid receptor upregulation, and increased medullary interleukin-6 and nerve growth factor (31).

However, prolonged bright light is analgesic. Hu et al. showed that bright light (3,000 lux, 2 h/day, 14 days) reduced formalin-induced hyperalgesia via the retinal ganglion cell-vLGN-PAG pathway, where vLGN/IGL projections to l/vlPAG inhibit GABAergic neurons and modulate pain regions (e.g., LC, RVM) to produce analgesia (32). Similarly, in female fibromyalgia patients and veterans with chronic low back pain, morning bright white light (>3,000 lux, 1 h/day) improved pain and function, linked to circadian modulation (19, 20, 33).

### 3.2 Green light

In a clinical study, Noseda et al. reported that visual exposure to white and red light intensified pain in migraineurs during acute migraine attacks, while green light elicited a markedly lesser exacerbation (34). A later trial showed that preventive green light (1–2 h/day, >10 weeks) reduced headache frequency, intensity, and duration in chronic migraine patients (21).

In other studies, green light also significantly reduced pain. Ibrahim et al. showed that green LED light (4 lux, 8 h/day, 5 days) produced antinociception in rats lasting up to 4 days (22). In other studies, Tang et al. found that green light (10 lux, 8 h/day, 6 days) relieved neuropathic and complete Freund's adjuvant (CFA)-induced inflammatory pain via the retina-vLGN-DRN pathway; inhibition blocked analgesia, activation relieved pain, penk-positive vLGN neuron knockdown/ablation reversed the effect, and vLGN-DRN projections inhibited pain responses (23).

Light also exerts analgesia by activating the circuit from the V2M to the ACC. Cao et al. found that green light (200 lux, 4 h/day, 7days) activates glutamatergic V2M (V2M^Glu^) projections to GABAergic ACC neurons, thereby suppressing ACC glutamatergic activity. Optogenetic or chemogenetic activation of this pathway in chronic constriction injury (CCI) and CFA models mimicked green light analgesia, while inhibition of V2M^Glu^-ACC projections abolished it (24).

Green light alleviates endometriosis-associated pain and slows lesion progression in mice via central endogenous opioid system activation, systemic noradrenaline increase, lesional beta-2 adrenergic receptor downregulation, cannabinoid receptor downregulation, and fibrosis attenuation (25). In monoiodoacetate -induced osteoarthritis rats, green light (200 lux, 6 h/day, 5 days) reduced secondary mechanical hyperalgesia in both sexes and restored weight-bearing in females, independent of peripheral nociceptor inhibition. Its analgesia was mediated by elevated serum N-acylglycine and blocked by G protein-coupled receptor 18 and cannabinoid receptor 1 antagonists receptor antagonists, implicating the endocannabinoid system (26). This effect appears sex-dependent, with greater fibromyalgia-like symptom relief in males (27).

### 3.3 Red light

Noseda et al. demonstrated that red light exposure exacerbates migraine pain (8), suggesting it favors pronociception over analgesia. Wiercioch-Kuzianik et al. observed that red light, when presented before painful stimuli, induced higher pain ratings than blue or green light, potentially due to its learned associations with failure, threat, and anger (9), suggesting that negative emotions may influence pain perception.

Khanna et al. reported that red LED (660 nm) produced time- and dose-dependent thermal hyperalgesia and mechanical allodynia in rats, effects reversible by RVM microinjection of the GABA-A antagonist bicuculline, implicating enhanced descending facilitation via the visual system–RVM axis that shifts pain modulation toward a pronociceptive state (10). Different colors modulate pain by regulating glutamatergic and GABAergic subpopulations in the vLGN. In a partial sciatic nerve ligation model, Wu et al. found that 7-day green light exposure (50 lux, 2 h/day, 7 day) reduced hyperalgesia, whereas red light (50 lux, 2 h/day, 7 day) exacerbated pain. Specifically, green light alleviated pain by activating vLGN glutamatergic neurons, while red light intensified pain by activating vLGN GABAergic neurons (11). There is limited direct clinical evidence regarding the analgesic effects of red light mediated through the visual system.

## 4 Local red and near-infrared LED-based PBM

Red and near-infrared wavelengths are commonly used for local PBM because they can penetrate superficial tissues more effectively than shorter visible wavelengths, although penetration depends on wavelength, tissue composition, pigmentation, beam geometry, and treatment dose (35). Unlike visual-light protocols, which commonly involve exposure for hours, local LED treatments are usually applied directly or close to the painful region for seconds to minutes. This section focuses on LED-based PBM treatment because LEDs differ from lasers in coherence, beam characteristics, and device design (36, 37). However, LEDs should not automatically be assumed to be safer or less powerful than lasers; safety depends primarily on irradiance, radiant exposure, treatment geometry, and thermal control (38).

### 4.1 Preclinical and clinical evidence

Preclinical studies support the analgesic effects of locally applied near-infrared LEDs in inflammatory, postoperative, and neuropathic pain models. In mice with complete Freund's adjuvant-induced inflammatory pain, plantar irradiation with a 950-nm LED for 25 s/day for 5 days reduced mechanical hypersensitivity (12). In a sciatic nerve-crush model, cutaneous treatment with a 950-nm LED for 32 s/day for 15 days attenuated mechanical hyperalgesia and was associated with lower tumor necrosis factor *α* (TNF-α) levels in the sciatic nerve and spinal cord (16). Near-infrared LED treatment has also reduced nocifensive responses in acute thermal and chemical pain assays (14).

Clinical evidence is more limited. A wearable 660-nm red-light device reduced neck pain and improved cervical range of motion, suggesting possible benefits for both pain and function (18). In a randomized controlled trial involving patients with temporomandibular disorders, combined 660- and 850-nm LED treatment for 20 min three times weekly for 2 weeks reduced pain but did not significantly improve mandibular mobility (17). These findings are encouraging, but the small number of trials and variability in treatment parameters prevent firm conclusions regarding condition-specific efficacy.

### 4.2 Proposed analgesic mechanisms

Several interacting mechanisms may contribute to the analgesic effects of local red and near-infrared LED treatment.

First, peripheral opioid signaling appears to play a role. In complete Freund's adjuvant-induced inflammatory pain, the effect of 950-nm LED treatment was blocked by intraplantar but not intrathecal naloxone, indicating the involvement of peripheral opioid receptors (12). In a plantar-incision model, 950-nm LED treatment recruited opioid-containing leukocytes to the injured tissue and promoted the release of endogenous opioid peptides (13).

Second, local LED treatment may modulate inflammatory and oxidative processes. In the inflammatory pain model, 950-nm LED treatment increased spinal interleukin-10 (IL-10) levels, enhanced peripheral antioxidant-enzyme activity, and reduced malondialdehyde concentrations (12). Following sciatic nerve crush, LED treatment was associated with lower TNF-α concentrations in both the sciatic nerve and spinal cord (16). These findings demonstrate associations with peripheral and spinal inflammatory changes, although they do not establish whether cytokine modulation is the primary cause of analgesia.

Third, PBM may reduce nociceptor sensitization through intracellular signaling pathways. In postoperative pain, 950-nm LED treatment modulated the L-arginine/nitric oxide (NO) pathway, potentially limiting pronociceptive NO signaling (13). Red and near-infrared LED treatment has also been associated with reduced reduced protein kinase A (PKA) and protein kinase C (PKC) activation (14, 15). Pharmacological experiments involving capsaicin-sensitive afferents further suggest that transient receptor potential vanilloid 1 (TRPV1)-expressing C-fibers contribute to the effect of near-infrared LED treatment (14). Nevertheless, these pathways may vary according to wavelength, tissue target, and pain model, and it remains unclear whether they represent primary photochemical effects or downstream consequences of reduced inflammation.

## 5 Green light as adjuncts to other therapies

**Figure 2:**
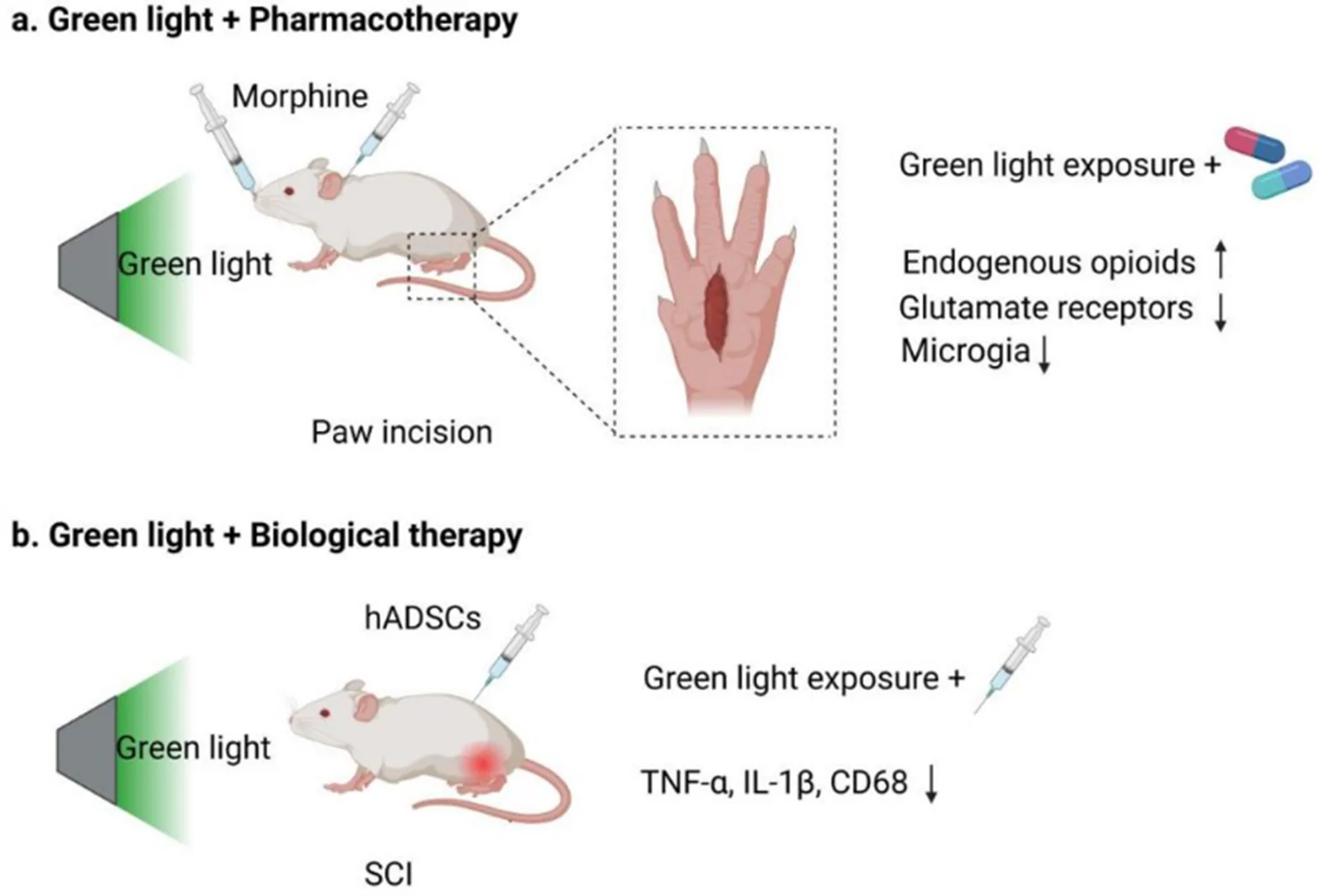
Combination strategies involving green light with adjunctive therapies. **(a)** In a rat paw incision model, perioperative green light enhances the antinociceptive effect of morphine. **(b)** Green light combined with human adipose-derived mesenchymal stem cells (hADSCs) alleviates pain by downregulating TNF-α, IL-1β, and CD68 expression.

PBM is more plausibly positioned as an adjunct than as a replacement for established pain treatment. Several studies have investigated the antinociceptive effects of combination of visual-light exposure. Combination strategies involving green light with adjunctive therapies are summarized in Figure 2.

In a rat paw-incision model, perioperative green-light exposure enhanced the antihyperalgesic effect of morphine compared with morphine alone or white light combined with morphine (39). This combined effect was associated with enhanced endogenous opioid signaling, reduced glutamate-receptor expression, and suppressed microglial activation. Green light also potentiated ibuprofen in male but not female rats, whereas no enhancement occurred with acetaminophen or gabapentin. These drug-and sex-specific findings indicate that the effect is not a nonspecific potentiation of all analgesics and warrant further replication.

Green-light exposure combined with human adipose-derived stem cells improved motor recovery, pain-related outcomes, and tissue repair after experimental spinal cord injury, possibly through modulation of microglial autophagy (40). Although promising, this finding is preclinical and cannot yet support clinical recommendations.

## 6 Discussion

Pain responses to light cannot be predicted from wavelength alone, because exposure route determines the initial target. Visual light acts through retinal, central, psychological, and circadian pathways; local red or near-infrared PBM acts on tissues, peripheral nerves, immune cells, and indirectly on spinal processing. These modalities are not mechanistically interchangeable.

Red light illustrates this distinction. Visual red light generally increases pain, whereas local red light reduces pain, as the same wavelength activates different targets depending on retinal versus tissue delivery. Visually perceived red may also carry learned threat associations, while local red light acts without conscious color perception. Future studies should test whether ocular shielding alters local red-light effects and whether expectancy contributes to visual responses.

White light is also context-dependent. Brief bright light facilitates pain, whereas repeated morning bright light may improve pain via circadian alignment, sleep, mood, or descending inhibition. Nighttime exposure should be considered separately, as circadian disruption and melatonin suppression may increase pain independently. Thus, timing, duration, irradiance, spectrum, and circadian state must be specified.

Green visual light produces relatively consistent antinociception in animals, involving vLGN-DRN, V2M-ACC, opioid, and endocannabinoid pathways. However, clinical evidence is limited: few human studies, small samples, and inherent blinding difficulties. Improvements may also reflect expectancy, avoidance of discomfort, reduced stimulation, or changes in sleep and mood. Green light is therefore a promising investigational tool, not an established treatment for fibromyalgia, osteoarthritis, or neuropathic pain; the strongest preliminary clinical evidence is for migraine.

Evidence for local red and near-infrared LED treatment is also uneven. Mechanistic studies suggest roles for peripheral opioid receptors, NO, PKA/PKC, TRPV1, inflammatory mediators, and oxidative stress, but most data are from rodents. Cytokine changes should not be interpreted as causal unless pathway blockade abolishes analgesia. Clinical studies suggest benefits in neck pain and temporomandibular disorders, but too few trials define optimal parameters.

Dosimetric heterogeneity complicates interpretation. Duration alone does not define dose, and contact application does not justify shorter treatment. Studies should report irradiance, radiant exposure, beam area, tissue distance, pulse parameters, session schedule, and calibration. For visual light, lux is insufficient for wavelength comparisons because it is visually weighted; spectral irradiance, photon flux, retinal exposure, ambient light, and circadian timing should also be reported. The biphasic dose response also warns against assuming that more irradiance or time produces greater analgesia.

Combination therapy evidence is promising but preliminary. Enhanced effects with morphine, ibuprofen, stem cells, cryotherapy, or exercise may reflect additivity, synergy, or pharmacological interaction, requiring factorial designs. Sex-specific effects merit attention, as sex, hormones, genotype, pigmentation, retinal function, expectations, and pain phenotype may all modulate response.

Thus, current evidence cannot support a general recommendation for any optimal wavelength, irradiance, exposure, or schedule; optimal parameters likely depend on route, target depth, tissue, and pain mechanism.

Future trials should be condition-specific, adequately powered, preregistered, and include credible sham controls. They should assess pain intensity and functional outcomes (physical function, sleep, quality of life, analgesic use, durability). Adverse events and treatment burden should be reported, especially as green-light protocols may require hours of daily exposure. Rather than seeking a single “analgesic wavelength,” research should define combinations of wavelength, route, dose, timing, and patient characteristics for specific pain mechanisms.

In conclusion, visual-light exposure and local LED PBM are promising but mechanistically distinct. Green light has the strongest preliminary support among visual color interventions, particularly for migraine; red and near-infrared LEDs may benefit selected localized conditions. Neither should replace standard care. Future work requires adequately powered, preregistered, condition-specific trials with credible controls and standardized reporting of wavelength, output, irradiance/illuminance, exposure, geometry, schedule, circadian timing, and adverse events. Dose-ranging studies are needed to identify combinations most likely to yield meaningful, durable benefit.
